# Cocaine-Induced Toxic Leukoencephalopathy: A Case Report

**DOI:** 10.7759/cureus.54574

**Published:** 2024-02-20

**Authors:** Shayet Hossain Eshan, Andranik Bedross, Gopika Chandra, Jose R Medina Inojosa, Shyam Chalise

**Affiliations:** 1 Internal Medicine, Ascension Saint Joseph Hospital Chicago, Chicago, USA; 2 Preventive Cardiology, Mayo Clinic, Rochester, USA

**Keywords:** substance use disorder (sud), altered mental status, levamisole, cocaine toxicity, cocaine use, toxic leukoencephalopathy

## Abstract

We present a case here where a 57-year-old South Asian male with disturbed mental status developed multifocal leukoencephalopathy, which we believe was caused by cocaine usage. Cocaine was detected in the urine toxicological sample. Non-acute CT head, with a follow-up brain MRI demonstrating hyperintensity of the T2 FLAIR signal corresponding to diffusion restriction throughout the whole supratentorial white matter, involving semiovale and subcortical U fibres in the occipital lobes as well as posterior frontal and parietal centrum. It was less likely that the patient had posterior reversible encephalopathy syndrome (PRES), which can potentially manifest similarly in a clinical and imaging context because there was no abrupt rise of blood pressure at presentation or during the patient's stay. Extensive examinations were conducted to exclude additional factors that may contribute to the patient's appearance, including autoimmune, vasculitis, and infectious diseases. Levamisole, a significant chemical that is frequently used to increase the volume of cocaine samples and has been linked to neuronal damage, should be examined in individuals who use cocaine and exhibit these kinds of clinical symptoms. The patient was prescribed 250 mg of methylprednisolone twice daily for five days after it was determined that cocaine-induced neuronal toxicity was the cause of his symptoms. Although no improvement was seen right away, over the course of the next few days, he did exhibit a gradual, albeit slight, improvement in his mental status while residing in the nursing home. It is crucial to comprehend the possible connection between cocaine usage, a commonly abused drug, and people exhibiting similar clinical symptoms. To have a better understanding of the pathophysiology and possible treatment approach, more research is necessary as there is now no recommended therapy regimen.

## Introduction

Cocaine-induced multifocal leukoencephalopathy is a rare condition that can result in serious long-lasting impairments. Clinical presentation varies significantly [1-5] and so does the prognosis, ranging from full recovery to fatal outcomes. Cocaine abuse is associated with several mechanisms of brain injury including convulsion, movement disorder, ischemic, hemorrhagic, and metabolic insults [2,4-6]. Results of magnetic resonance imaging (MRI) can also vary significantly and generally involve the subcortical white matter [1-8]. Multifocal leukoencephalopathy has been infrequently reported in rare cases of cocaine use. In addition to the drug, levamisole is found as a common adulterant in many cocaine to increase the volume and has been implicated as a culprit for neuronal toxicity [1-4, 9,10]. We present a case of cocaine-induced multifocal leukoencephalopathy and the challenges of treatment. 

## Case presentation

A 57-year-old South Asian male with a past medical history of hypertension, hyperlipidemia, cocaine use disorder, tobacco use disorder, and recent ischemic stroke a month prior to presentation localized in the right aspect of the medulla, was brought into the hospital from home by his son with altered mental status. He was found unconscious at home by neighbors and he was last known to be well about 30 hours before presenting to the emergency department. The patient’s son and neighbors provided a history of recent cocaine use. 

Upon admission, he was hemodynamically stable with vital signs showing blood pressure 125/75 mmHg, pulse of 64 beats per minute, temperature of 98.8 °F, respiratory rate of 18 per minute, and oxygen saturation of 98% on room air. Physical examination was unremarkable except for lethargy and oriented to only his name. Initial laboratory tests included complete blood count (CBC), comprehensive metabolic panel (CMP), serum magnesium, serum phosphorus, arterial blood gas (ABG), blood alcohol level, syphilis screen, and thyroid stimulating hormone (TSH), as shown in Table 1. Laboratory tests were largely unrevealing to the clinical presentation.

**Table 1:** Showing Complete Blood Count (CBC), Comprehensive Metabolic Panel (CMP), serum magnesium, serum phosphorus, Thyroid-stimulating hormone (TSH), syphilis screen and arterial blood gas (ABG) CBC= Complete Blood Count, WBC= White blood cells, CMP=Comprehensive Metabolic Panel, BUN=Blood urea nitrogen,  AST=Aspartate Aminotransferase, ALT=Alanine Aminotransferase, TSH=Thyroid-stimulating hormone, ABG=Arterial blood gas, pCO2= Partial pressure of carbon dioxide, pO2=Partial pressure of oxygen

Laboratory test	Result	Reference
Hemoglobin	13.2 g/dl	13-17 g/dl
Hematocrit	38.9%	38.6-49.2%
Platelet	197 k/mm cu	150-450 k/mm cu
White blood cells (WBC)	6.2 k/mm cu	4-11 k/mm cu
Sodium	142 mmol/L	133-144 mmol/L
Potassium	3.8 mmol/L	3.5-5.2 mmol/L
Chloride	106 mmol/L	98-107 mmol/L
Bicarbonate	30 mmol/L	21-31 mmol/L
Blood urea nitrogen (BUN)	16 mg/dL	7-25 mg/dL
Creatinine	0.96 mg/dL	0.7-1.3 mg/dL
Glucose	106 mg/dL	70-99 mg/dl
Aspartate Aminotransferase (AST)	15 IU/L	13-39 IU/L
Alanine Aminotransferase (ALT)	19 IU/L	7-52 IU/L
Total bilirubin	0.4 mg/dL	0.0-1.0 mg/dL
Albumin	4.2 g/dL	3.5-5.7 g/dL
Calcium	9.4 mg/dL	8.6-10.3 mg/dL
Magnesium	2.2 mg/dL	1.6-2.6 mg/dL
Phosphorus	4.2 mg/dL	2.5-4.5 mg/dL
Thyroid-stimulating hormone (TSH)	1.63 uIU/mL	0.27-4.2 uIU/mL
Syphilis screen (total IgG/IgM)	Non-reactive	Non-reactive
Blood alcohol level	<10 mg/dL	0-10 mg/dL
Arterial blood gas (ABG)		
pH, arterial	7.41	7.35-7.45
Partial pressure of carbon dioxide (pCO2), arterial	39.6 mmHg	35-45 mmHg
Partial pressure of oxygen (pO2), arterial	97.6 mmHg	85-102 mmHg
Carbon Monoxide	2.1 %	0.5-1.5%
Methemoglobin	0.6%	0-1.5%

**Figure 1:**
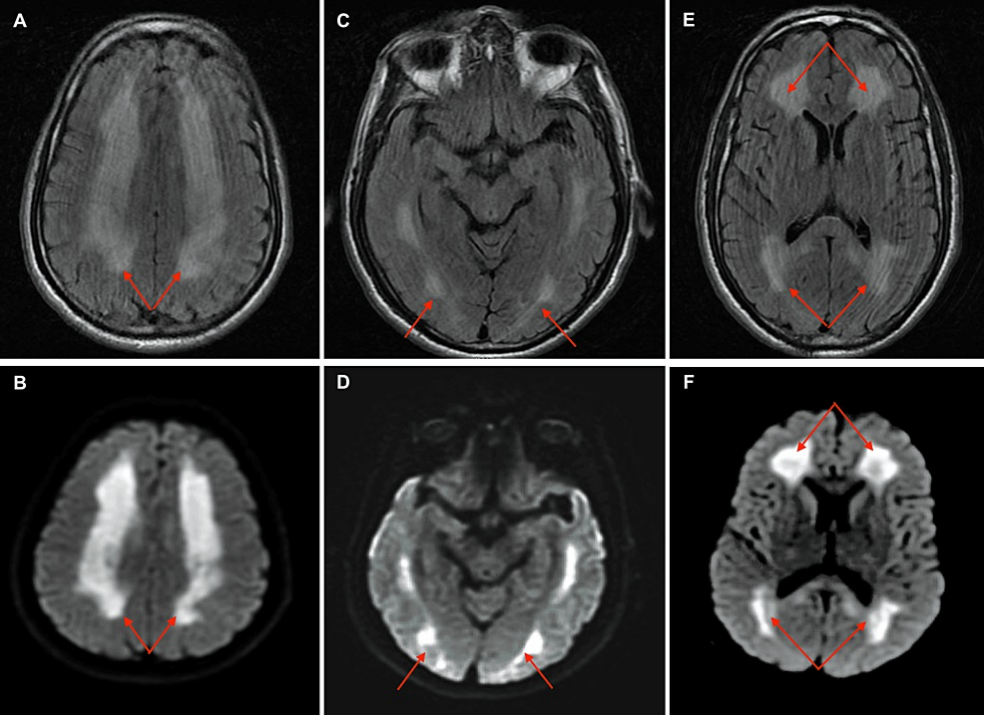
MRI brain showing T2 fluid-attenuated inversion recovery (FLAIR) signal hyperintensity with corresponding restricted diffusion throughout entire supratentorial white matter (red arrows) insult suggestive of multifocal leukoencephalopathy. No acute focal infarct, hemorrhage, mass effect, or hydrocephalus. Images A, C, and E represent T2 fluid-attenuated inversion recovery (FLAIR) hyperintensities over posterior frontal and parietal centrum semiovale, subcortical U fibers in the occipital lobe and white matter in basal ganglia respectively. Images B, D, and F demonstrate corresponding diffusion restriction on Diffusion-weighted imaging (DWI).

Computed Tomography (CT) scan of the head without contrast as well as CT angiography head and neck was unrevealing. Urine toxicology was only positive for cocaine. Magnetic Resonance Imaging (MRI) brain was performed and showed restricted diffusion corresponding to T2 fluid-attenuated inversion recovery (FLAIR) signal hyperintensities throughout the entire supratentorial white matter involving posterior frontal and parietal centrum semiovale and subcortical U fibers in the occipital lobes consistent with multifocal leukoencephalopathy (Figure 1). 

A lumbar puncture was performed and showed normal opening pressures, clear fluid, and no leukocytes. Cerebrospinal fluid (CSF) microbiological testing forherpes simplex virus type 1 (HSV-1), herpes simplex virus type 2 (​​​HSV-2), John Cunningham (JC) virus, cryptococcal antigen, meningoencephalitis panel, and tuberculosis was negative, without evidence of bacterial growth in CSF Gram staining or culture. Further workup for human immunodeficiency virus (HIV), syphilis, Epstein-Barr virus (EBV), autoimmune, and vasculitis including antinuclear antibody and antineutrophil cytoplasmic antibodies were also negative. A normal level of 302 pg/mL vitamin B12 was noted thus ruling out vitamin B12 insufficiency can also show up with similar imaging findings and clinical manifestations, such as behavioral disorders, neuropathy, dementia, and subacute combined degeneration. Electroencephalogram showed no evidence of seizure-like activity and diffuse bihemispheric slowing, a finding consistent with the diffuse encephalopathic process. 

As no other potential causes for the patient's encephalopathy were found, it was deemed that his current presentation was secondary to cocaine use. He was placed on intravenous methylprednisolone 250 mg twice a day for five days because supportive care and steroids have been associated with varying responses in some of the case reports. Many of the case reports in the literature utilized 1 g of methylprednisolone daily for 3-5 days, and some of them employed varying doses and durations of the steroid [1,2,6, 8-12]. This led to a range of responses, from full recovery to death. The decision was made to take a more cautious approach to the administration of 250 mg twice daily of IV methylprednisolone because there are no guidelines in the treatment of these patient instances. Corticosteroids, especially when taken in large amounts, can cause neurologic and mental symptoms.

His mentation remained poor during hospitalization and an MRI brain was repeated, revealing stable lesions similar to prior imaging. He underwent percutaneous gastrostomy tube placement and was sent to a nursing home for continued supportive care as toxic leukoencephalopathy is known to improve potentially over the course of months. After 45 days post-discharge, a follow-up call was made to the son who reported that the patient continues to be in the nursing home and has made some progress in his mentation. 

## Discussion

The clinical spectrum of cocaine-induced toxic leukoencephalopathy presentation varies widely with various symptoms ranging from confusion, ataxia, inattention, hyperreflexia, psychomotor retardation, stupor, spasticity, weakness, hemiparesis, headache, fever, delirium or coma due to its particular involvement of white matter tracts [1-5]. Cocaine-induced toxic leukoencephalopathy has been less frequently reported compared to drugs such as heroin or other opioids. No difference has been noted between the duration or route of cocaine administration and the development of toxic leukoencephalopathy [2]. Diagnosing cocaine-induced leukoencephalopathy requires a high level of suspicion given the rarity of such cases and the broad range of non-specificity of symptom presentation, especially with the use of new synthetic drugs which are undetectable in regular drug screens. Other causes of leukoencephalopathy need to be excluded including but not limited to infectious, inflammatory, autoimmune, genetic disorders, and posterior reversible encephalopathy syndrome [2]. 

The pathophysiological mechanism is complex including neuroinflammation, ischemia/hypoxia, and direct neurotoxicity largely linked to axonal damage [2, 4-6]. This can result in various MRI findings involving white matter with or without sparing of subcortical U fiber best seen on T2 FLAIR MRI. In some cases, there was the involvement of semioval centers and occipital fibers of the radiating crowns and inner capsules [1-7]. Balo’s concentric sclerosis on post-gadolinium contrast T1-weighted images can also be seen [8]. In our case, the MRI brain there is restricted diffusion with corresponding T2 FLAIR demonstrated white matter involving posterior frontal and parietal centrum semiovale and subcortical U fibers in the occipital lobes (Figure 1). 

In addition to the drug itself, there have been several associations of levamisole, an adulterant found in up to 80% of cocaine samples, behind the features seen in cocaine-induced leukoencephalopathy. Levamisole has immune-stimulating properties through monocyte chemotaxis and enhancing macrophage functions which could be responsible for possible immune-mediated neuronal toxicity [1-4,9,10]. It might also potentiate the euphoric effects of cocaine by inhibiting dopamine reuptake and forming amphetamine-like metabolites [3]. Unfortunately, levamisole was not tested for our patient. 

The challenge lies in the treatment as there has yet to be an official consensus on clinical management. Proposed treatment ranges from symptomatic management to pulse steroid therapy with or without intravenous immunoglobulin or plasma exchange with varying outcomes from complete clinical recovery to fatalities. In each subset, the duration of recovery is highly variable. 

Methylprednisolone was the steroid used in most case reports in the literature with complete to partial recovery in many patients within 2 weeks to 3 months [1,2,6,8-10]. Some cases failed to show any improvement on steroids [11] with fatal outcomes in the remaining [12]. Targeted immunosuppression with IVIG, plasma exchange, and cyclophosphamide in conjunction with steroids also yielded variable responses with improvement varying from complete to partial recovery within 24 months period [3,4,7,13]. On the contrary, one of the cases did show worsening symptoms after the use of steroids with plasmapheresis, and hence cyclophosphamide was added due to the refractoriness of the symptoms with no additional benefit [3]. 

Supportive treatment also yielded variable outcomes with clinical recovery to fatal outcomes [5,14]. Treatment with co-enzyme Q10, dantrolene, and bromocriptine with complete recovery in 8 weeks in one of the cases [15]. 

When interpreting brain imaging in a clinical setting such as this, it's crucial to keep in mind that Posterior Reversible Encephalopathy Syndrome (PRES) can also manifest with similar MRI results but involvement of subcortical white matter is uncommon [16]. PRES also presents with an acute increase in blood pressure with the usual peak of systolic blood pressure between 170 - 190 mmHg in about 70 percent of cases, although normal or slightly elevated blood pressure with PRES has been reported [17]. Many drugs including prescription medications such as immunosuppressive, immunomodulatory, and chemotherapeutic as well have drugs of abuse including cocaine, amphetamine, kratom, and lysergic acid amide have been reported to be linked with the development of PRES [18-20]. Given the overlap of clinical symptoms and imaging findings of PRES and toxic leukoencephalopathy, differentiating the two can be challenging but in the absence of acute blood pressure elevation and predominance of subcortical U fiber involvement on MRI, our patient finding is more consistent with cocaine-induced toxic leukoencephalopathy. 

Our patient’s mental status remained poor during the hospitalization even after receiving methylprednisolone 250 mg twice daily for 5 days and supportive treatment. At the nursing home following discharge, his mental status improved gradually over 45 days, and is currently responding to commands but continues to be non-verbal. 

## Conclusions

Cocaine-induced leukoencephalopathy, a rare and deadly neurological disorder that damages the white matter and causes a range of neurological symptoms, is connected to cocaine usage. Given its poor prognosis and, in certain cases, potentially swiftly deadly conclusion, it is imperative that healthcare professionals become more knowledgeable about this disorder. Adulterants like levamisole, which are added to cocaine to increase its volume and have been connected to a potential cause of such clinical manifestation, further complicate the problem. The lack of recommended treatment guidelines and variable responsiveness to some proposed treatment approaches poses a barrier to the therapy of cocaine-induced leukoencephalopathy. To further our knowledge of cocaine-induced leukoencephalopathy and improve patient care, more research is required to determine the mechanism, available treatments, and long-term effects of this disorder. Levels of adulterants such as levamisole might provide further reflections on categorizing the offender, whether it is the drug of abuse, adulterant, or combination of both.
